# Increased gene expression variability in *BRCA1*-associated and basal-like breast tumours

**DOI:** 10.1007/s10549-021-06328-y

**Published:** 2021-07-21

**Authors:** George A. R. Wiggins, Michael A. Black, Anita Dunbier, Arthur E. Morley-Bunker, kConFab Investigators, John F. Pearson, Logan C. Walker

**Affiliations:** 1 Department of Pathology and Biomedical Science, University of Otago, Christchurch, New Zealand; 2 Department of Biochemistry, University of Otago, Dunedin, New Zealand; 3 Sir Peter MacCallum Department of Oncology, University of Melbourne, Melbourne, VIC Australia; 4 Research Department, Peter MacCallum Cancer Center, Melbourne, VIC Australia; 5 Biostatistics and Computational Biology Unit, University of Otago, Christchurch, New Zealand

**Keywords:** Gene expression variability, *BRCA1*, *BRCA2*, Breast cancer, Microarray, RNAscope

## Abstract

**Purpose:**

Inherited variants in the cancer susceptibility genes, *BRCA1* and *BRCA2* account for up to 5% of breast cancers. Multiple gene expression studies have analysed gene expression patterns that maybe associated with *BRCA12* pathogenic variant status; however, results from these studies lack consensus. These studies have focused on the differences in population means to identified genes associated with *BRCA1/2*-carriers with little consideration for gene expression variability, which is also under genetic control and is a feature of cellular function.

**Methods:**

We measured differential gene expression variability in three of the largest familial breast cancer datasets and a 2116 breast cancer meta-cohort. Additionally, we used RNA in situ hybridisation to confirm expression variability of *EN1* in an independent cohort of more than 500 breast tumours.

**Results:**

*BRCA1*-associated breast tumours exhibited a 22.8% (95% CI 22.3–23.2) increase in transcriptome-wide gene expression variability compared to BRCAx tumours. Additionally, 40 genes were associated with *BRCA1*-related breast cancers that had ChIP-seq data suggestive of enriched *EZH2* binding. Of these, two genes (*EN1* and *IGF2BP3*) were significantly variable in both *BRCA1*-associated and basal-like breast tumours. RNA in situ analysis of *EN1* supported a significant (*p* = 6.3 × 10^−04^) increase in expression variability in *BRCA1*-associated breast tumours.

**Conclusion:**

Our novel results describe a state of increased gene expression variability in *BRCA1*-related and basal-like breast tumours. Furthermore, genes with increased variability may be driven by changes in DNA occupancy of epigenetic effectors. The variation in gene expression is replicable and led to the identification of novel associations between genes and disease phenotypes.

**Supplementary Information:**

The online version contains supplementary material available at 10.1007/s10549-021-06328-y.

## Introduction

Gene expression profiles have been used extensively in the study of cancer development, treatment response and prognosis. In particular, gene expression signatures have been developed to classify tumour subtypes [1, 2], predict response to endocrine treatment [3, 4], indicate prognosis [3, 5, 6] and predict tumour recurrence [4]. The development of these signatures has relied on capturing a change in average expression between biological groups (e.g. poor responders versus good responders) and subsequently validating these results in independent datasets. The variability in gene expression levels (e.g. across samples) is often ignored and is generally only considered in terms of the impact it has on statistical power. However, gene expression variability has been shown to be under genetic control and important to cellular function [7–11]. Gene expression variability has been used to evaluate transcriptomic data in human disease and development [12–15]. By isolating individual embryonic cells, researchers have shown that gene expression variability provides insights into gene regulation that is essential throughout embryonic development [12]. Differences in gene expression variability have also been investigated to improve our understanding of cancer biology. Although a few studies have been reported to date, this approach has led to the identification of a pan-cancer gene set [16], a classifier for chronic lymphocytic leukaemia [17] and synthetic lethal genes in *BRCA2*-associated ovarian tumours [18]. These studies measured gene expression variability of whole-transcriptome data generated from microarray or RNA-sequencing platforms. Two of these studies highlighted the utility of measuring global gene expression variability from microarray data to classify leukaemia subtypes [17] and to identify a 48 gene set as a predictive marker of cancer metastatic potential and patient survival [16]. One study identified genes from RNA-sequencing data with potential synthetic lethal interactions with *BRCA2* in ovarian cancer [18]. Genetic variants in *BRCA1* and *BRCA2* predispose women to breast and ovarian cancers and are believed to contribute to 5–10% of all breast cancers and 20–40% of familial breast cancers [19, 20]. Despite representing distinct tumour types, there has been limited success in identifying gene expression profiles related to *BRCA1* and *BRCA2* pathogenic variants in either tumour [3, 21–24] or normal tissue [25–28]. Across all studies, consensus on altered genes and pathways has been poor, with gene expression profiles influenced by study design rather than variant classification [29]. For example, early studies were confounded by differences in oestrogen receptor (ER) status of *BRCA1*-associated tumours compared to sporadic [21]. The ER status of breast tumours was later identified to be a major driver of gene expression changes [3]. These studies have also overlooked the variability in gene expression and whether these phenotypes are associated with the presence of a pathogenic variant.

In this study, we investigate transcriptomic data across multiple breast tumour datasets using differential variability analysis to identify genes that are associated with *BRCA1* and *BRCA2* pathogenic variant status and with different tumour subtypes. We also utilised RNA in situ hybridisation as an orthogonal approach to validate inter-tumour expression variability of a candidate gene in an independent cohort of breast tumours.

## Methods

### Data collection

For gene expression variability analyses, we included any microarray dataset containing gene expression profiles on at least 50 breast tumours, of which > 25 samples were either *BRCA1*- or *BRCA2*-associated breast tumours (Table 1). Raw data were acquired through GEO (https://www.ncbi.nlm.nih.gov/geo/) for three datasets (GSE19177, GSE27830 and GSE49481) generated on Illumina, Affymetrix and Agilent microarray platforms, respectively [23, 30, 31]. Raw intensities collected on the Illumina and Agilent arrays were normalised using quantile normalisation. Additionally, for the Agilent array only the intensity values (CY5 channel) corresponding to breast tumour RNA were considered. Raw intensities obtained on the Affymetrix arrays were normalised using the RMA algorithm [32].

**Table 1:** Breast cancer datasets

Dataset	Repository (GEO)	No. of samples (BRCA1/2)	Array platform
Waddell	GSE19177	74 (49)	Illumina Human 6v.2
Nagel	GSE27830	129 (53)	Affymetrix HG U133 plus 2.0
Larsen	GSE49481	253 (55)	Agilent SurePrint G3
Meta-cohort	–	2116	Affymetrix HG-U133 series*

*Includes Affymetrix U133A, U133plus2 and U133A2

In addition, a retrospective microarray dataset of well-curated breast cancer gene expression profiles [33] was utilised for a subtype-centric analysis [33]. Briefly, all samples were profiled on one of the Affymetrix U133A, U133A2 or U133plus2 GeneChip arrays. For consistency, only probes common to all arrays were retained. Individual arrays were normalised using the RMA method and batch effects were corrected using the COMBAT method [34]. The resulting dataset consisted of 2116 breast tumours and 22,268 probes.

Lastly, we accessed the METABRIC dataset through cbioportal (www.cbioportal.org/), in order to investigate DV genes relationship with molecular features.

### Transcriptome-wide gene expression variability analysis

Transcriptome-wide gene expression variability was compared between *BRCA1*-associated tumours and non-*BRCA1/2* (BRCAx) hereditary breast tumours, *BRCA2*-associated tumours and BRCAx and Basal-like and non-basal (luminal A, luminal B, HER2 and normal-like) tumours. To assess transcriptome-wide changes, tumour type-specific standard deviation (SD), coefficient of variance (CV) and median absolute deviation (MAD) were calculated for each gene. Pairwise linear regression models were calculated between tumour groups for each gene-specific statistics (i.e. SD, CV, MAD and mean) and the resulting *β* (slope coefficient) was compared to a model of tumour equity (*β* = 1). We defined the difference in expression variability between tumour groups as the percentage change in the slope coefficient compared to model of tumour equity (1 – *β* × 100). Additionally, pairwise polynomial regression was performed to investigate non-linear relationships between tumour-specific statistics.

### Differential expression variability analysis

Genes were considered to exhibit differential expression variability (hereafter referred to as differentially variable—DV-genes) if the spread of expression values differed significantly between two tumour groups. For these analyses, the same tumour comparison was made as in the transcriptome-wide analyses (i.e. *BRCA1* versus BRCAx, *BRCA2* versus BRCAx and basal-like versus non-basal). Robust measures of spread were used to avoid spuriously large differences caused by outlying expression values. Spread was quantified by the median absolute deviation (MAD) and groups were compared by the Brown–Forsythe method [35], essentially an ANOVA on expression values about their medians, with *p*-values adjusted for multiple testing [36] was used to determine significance. Ratios of MADs with 95% confidence intervals [37] were used to quantify changes in spread between groups.

### Breast cancer tissue microarrays

Women diagnosed with breast cancer were identified from 1800 families recruited into the Kathleen Cunningham Consortium for Research into Familial Breast Cancer (kConFab) [38]. For inclusion in this consortium, families must have a strong family history of breast and/or ovarian cancer, or be known to be segregating a germline variant in genes such as *BRCA1* and *BRCA2* (see www.kconfab.org for recruitment criteria). Breast cancer cases on tissue microarrays (TMAs) were verified from pathology reports. Ethics approval was obtained from the HREC at the Peter MacCallum Cancer Centre (97/27) and through the University of Otago Human Ethics Committee (H14/088). Informed consent at study entry was obtained from all participants, allowing access to medical/treatment reports, blood collection and tumour tissue collections. For deceased participants proxy consent was obtained from the next of kin. Where applicable, cause of death was verified from a death certificate, doctor or hospital medical records. Treatment and medical notes were accessed through physicians, hospitals, laboratories and State Cancer Registries.

Confirmation of a participant’s germline mutation status was performed using a variety of sequencing platforms in the Molecular Pathology NATA-accredited clinical laboratory. Variants were assigned a class C4–C5 (pathogenic) mutation status according to a 5-tier clinical classification introduced by ENIGMA (http://www.enigmaconsortiumorg).

### RNA in situ hybridisation (RNAscope)

The mRNA expression level for *EN1* was investigated using the RNAscope 2.0 BROWN assay (Advanced Cell Diagnostics, Inc.) following manufacturers’ instructions. Additionally, mRNA levels of *PPIB* and the bacterial gene *dapB* were used as positive and negative controls, respectively. Briefly, TMA sections were deparaffinised in a series of xylene and 100% ethanol steps. Sections were subjected to a series of pre-treatments before each section was incubated with target or control probes for 2 h at 40 °C in a HybEZ™ Oven. Probe signal was amplified through a series of amplification steps and colour development was done using diaminobenzidine (DAB).

### Scoring/counting signals

All TMA cores were scored for abundance of *PPIB* and *DapB* signals where a positive signal was assessed as a brown punctuate dot within a cell. Supplementary Table 1 describes the criteria for each score. Briefly, a score of ‘0’ described an absence of signal, ‘0.5’ was a weak stain with < 30% of cells having a signal, ‘1’ was a modest stain with > 30% of cells with a positive signal, ‘2’ was considered moderate staining with a greater number (4–9) of signals per cell, ‘3’ was strong staining with the presence of signal clustering in < 10% of cells and ‘4’ was intense staining with signal clustering in > 10% of cells.

All TMA sections were scanned with the Aperio Scanner and digital scans were used to quantify mRNA signals using the LEICA RNA ISH algorithm (Leica Microsystems GmbH, Germany). Briefly, the algorithm was trained on a selection of cores from each TMA and across a range of genotypes. These settings were applied to all the cores stained with probes targeting *EN1* RNA.

### Transcription regulator enrichment analysis

To identify DNA binding elements that were overrepresented in a set of DV genes, the *TFEA.ChIP* package in R was used [39]. ChIP-Seq datasets were obtained through the TFEA.ChIP github page (https://github.com/LauraPS1/TFEA.ChIP_downloads). Briefly, datasets from the ENCODE Consortium, DeMap and individual GEO database were included in the analysis, and gene sequences were annotated based on transcription regulator binding within 1 kb of each gene.

### Statistical analysis

R (version 3.6.1) was used to normalise microarrays and perform all statistical analyses. For microarrays on the Affymetrix platform, the RMA normalisation [32] from the *affy* package was applied, whilst for each of the Agilent and Illumina arrays, the quantile normalise method from the *limma* package was used [40]. The *lawstat* package was used to calculate Brown–Forsythe test. To account for multiple testing, *p-*values were adjusted using the Benjamini–Hochberg procedure [36].

## Results

### Transcriptome-wide gene expression variability

**Fig. 1:**
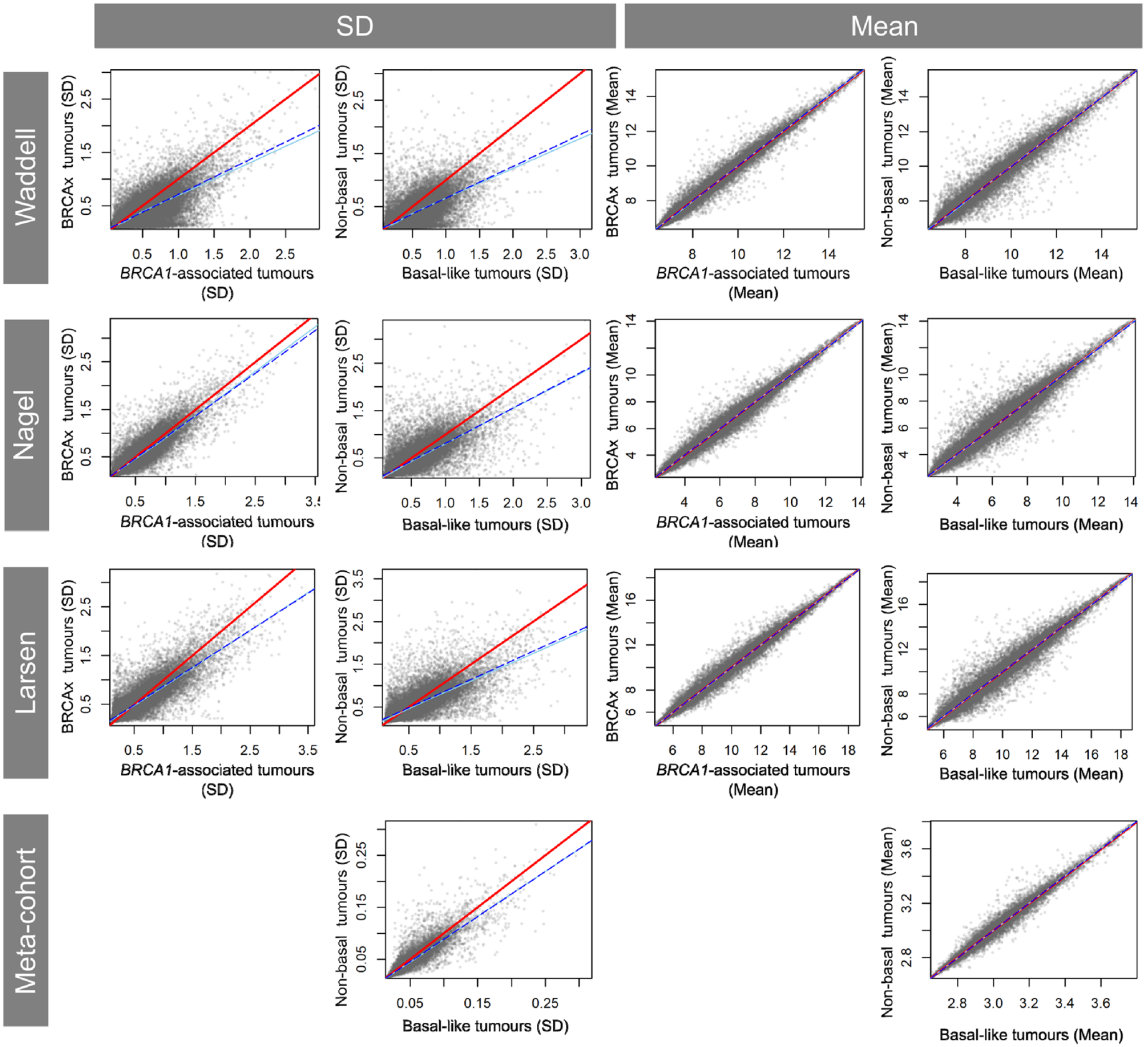
Transcriptome-wide gene expression variability in breast tumours. *BRCA1*-associated and basal-like breast tumours each show greater gene-specific standard deviations compared to BRCAx and non-basal tumour, respectively. Additionally, global gene-specific means between tumour groups are depicted. A model of equity (red line) was compared to the linear model (blue dashed line) and polynomial regression (sky blue line)

Differences in transcriptome-wide gene expression variability between familial breast cancer groups (*BRCA1, BRCA2* and BRCAx) were quantified by comparing tumour-specific measurements of variability (SD, CV and MAD). *BRCA1*-related breast tumours share multiple biological properties with sporadic basal-like breast cancer [41], therefore we also assessed gene expression variability differences between the basal and non-basal breast tumour subtypes. *BRCA1*-associated and basal-like breast tumours displayed greater transcriptome-wide variability compared to non-*BRCA1* and non-basal-like (luminal A, luminal B, HER2 and normal-like) tumours (Fig. 1, Supplementary Fig. 1). Using gene-specific standard deviations, *BRCA1*-associated breast tumours had a 22.8% (95% CI 22.3–23.2) increase in gene expression variability. Increased variability in *BRCA1*-associated breast tumours was also observed in linear models of gene-specific CVs (25.0%, 95% CI 24.5–25.6) and MADs (32.4%, 95% CI 31.9–33.0). Similarly, basal-like tumours had an average 28.2% (95% CI 27.7–28.7) greater transcriptome-wide expression variability compared to non-basal tumours. Gene expression variability in *BRCA2*-associated tumours was inconsistent, with the Waddell dataset showing comparable variability with BRCAx tumours (Supplementary Fig. 2), but the Larsen dataset showing only a modest 11.1% (95% CI 10.6–11.6) increase in variability in *BRCA2*-associated tumours compared to BRCAx tumours. The Nagel dataset had too few *BRCA2*-associated tumours to reliably estimate transcriptome-wide expression variability. In contrast to gene expression variability, no changes were observed in transcriptome-wide mean expression for any tumour groups tested.

### Differential expression variability in breast tumours

**Fig. 2:**
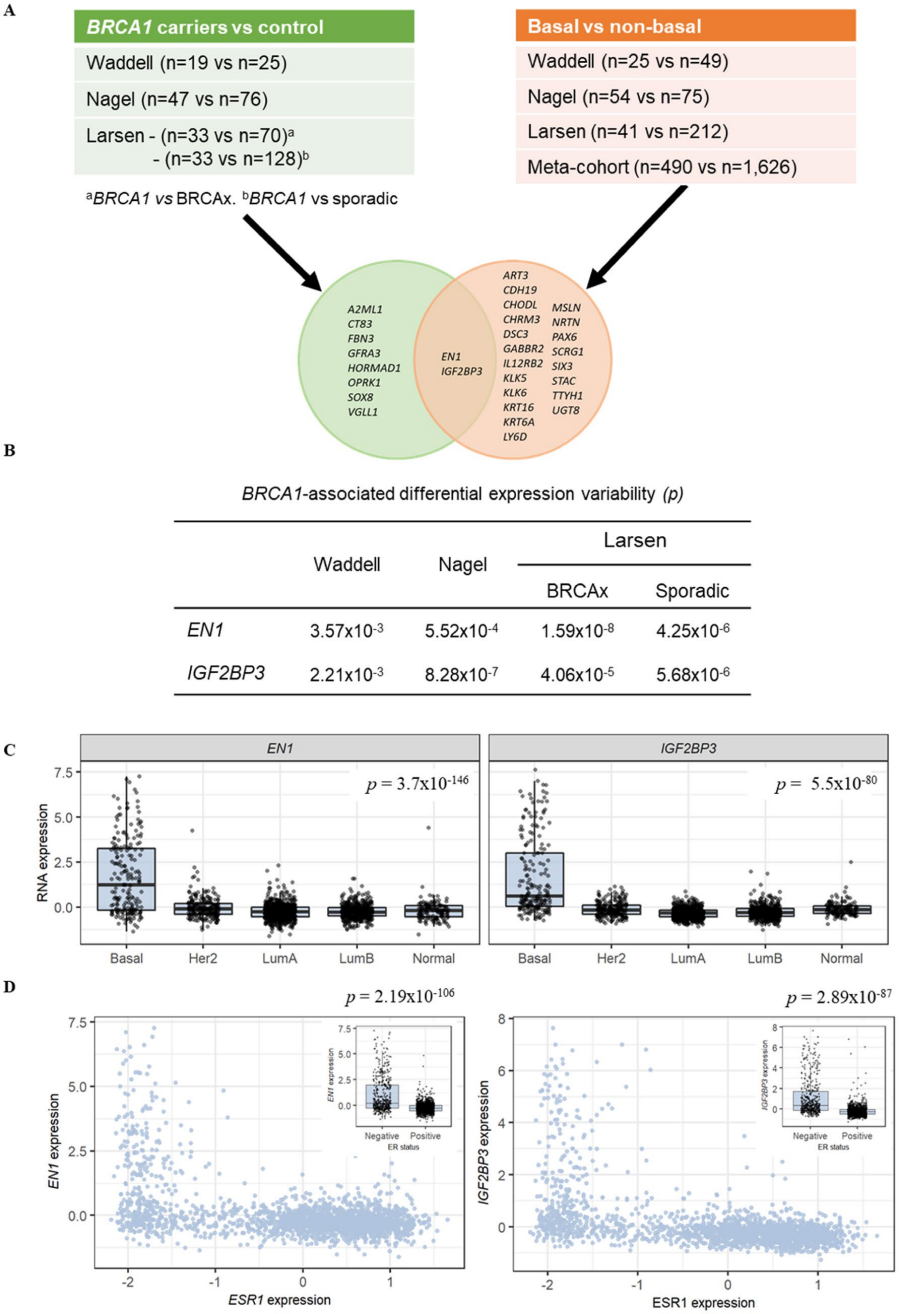
Identification of *BRCA1*-associated candidate gene(s). **A** Intersection between the consensus *BRCA1*-associated variable genes (green) and basal-like-associated variable genes (orange). **B** Significances of differential variability genes that intersected both analyses from **A**. **C** *EN1* (left) and *IGF2BP3* (right) expression in the METABRIC dataset stratified by intrinsic subtype. *p*-values were calculated by the Brown–Forsythe test comparing basal-like tumours to all others. **D** Correlation of *EN1* (left) and *IGF2BP3* with *ESR1* expression and ER status (insert). All *p*-values were estimated using the Brown–Forsythe test

As *BRCA1*-associated and basal-like breast tumours displayed greater transcriptome-wide expression variability, it was of interest to identify specific genes that had altered variability between tumour types. After adjusting for multiple comparisons, 503 and 337 genes were found to be significantly differentially variable between *BRCA1*- and BRCAx-associated breast tumours in the Nagel and Larsen datasets, respectively. There were 40 DV genes that were consistent in both the Nagel and Larsen datasets (Table 2). The directionality of the gene expression variability was consistent for all 40 DV genes. Additionally, 36 of the 40 *BRCA1*-associated DV genes were also DV between *BRCA1*-associated and sporadic breast tumours. A total of 185 basal-like-associated DV genes were identified across the four breast tumour datasets, with 184/185 (99.5%) consistent in direction. Analysis of the meta-cohort data, which comprised 2116 samples, found 58% of all genes to be DV. To identify candidate genes for in situ expression analysis, we compared the top 1.5% most significant DV genes across all datasets and between subtype and genotype analyses (Fig. 2A). A total of 10 *BRCA1*-associated and 22 basal-like-associated variable genes were identified in all datasets at 1.5% threshold. *EN1* and *IGF2BP3* were variably expressed in both *BRCA1*-associated and basal-like breast tumours (Fig. 2B).

**Table 2:** 40 consensus significant *BRCA1*-associated variable genes

Gene symbol	Larsen			Nagel		
	MAD ratio (95% CI)	*p* value	Adjusted *p* value	MAD ratio (95% CI)	*p* value	Adjusted *p* value
*A2ML1*	100.59 (33.91–298.4)	4.85E−06	2.58E−03	8.65 (2.65–28.24)	4.66E−04	3.73E−02
*AFAP1-AS1*	3.9 (0.71–21.49)	2.81E−04	2.51E−02	12.25 (3.97–37.76)	4.73E−04	3.76E−02
*CLOCK*	4.23 (1.71–10.48)	3.43E−04	2.71E−02	4.39 (1.79–10.76)	1.98E−04	2.45E−02
*COL22A1*	20.66 (8.32–51.28)	1.12E−06	1.06E−03	29.71 (10.84–81.43)	2.39E−09	2.40E−05
*CT83*	21.9 (4.13–116.2)	4.87E−07	6.09E−04	127.09 (45.43–355.5)	2.37E−04	2.63E−02
*CYP27C1*	6.11 (1.94–19.29)	1.03E−04	1.45E−02	10.23 (3.52–29.76)	1.28E−05	5.06E−03
*DLX2*	0.1 (0.03–0.33)	1.53E−04	1.87E−02	0.1 (0.04–0.29)	3.36E−05	8.76E−03
*ELOVL4*	4.2 (1.41–12.5)	1.30E−04	1.69E−02	4.12 (1.58–10.71)	1.29E−05	5.06E−03
*EN1*	10.77 (3.93–29.48)	1.59E−08	5.63E−05	12.65 (4.88–32.76)	5.52E−04	3.98E−02
*FBN3*	21.06 (8.28–53.56)	5.86E−06	2.68E−03	4.57 (1.88–11.1)	2.93E−07	5.88E−04
*GABBR2*	54.13 (19.58–149.6)	6.70E−04	3.73E−02	42.2 (15–118.69)	1.45E−05	5.29E−03
*GFRA3*	4.03 (1.08–15.14)	2.39E−04	2.29E−02	4.98 (1.79–13.85)	5.88E−04	4.06E−02
*HORMAD1*	11.43 (2.63–49.77)	3.40E−04	2.70E−02	504.73 (191.28–1332)	5.95E−05	1.21E−02
*HRCT1*	7.72 (2.9–20.57)	1.49E−09	1.05E−05	15.55 (5.69–42.49)	4.13E−06	2.86E−03
*IGF2BP3*	14.05 (4.07–48.47)	4.06E−05	8.45E−03	25 (7.93–78.83)	8.28E−07	1.04E−03
*IL12RB2*	11.86 (3.76–37.36)	8.06E−04	4.12E−02	27.08 (10.87–67.45)	4.57E−04	3.69E−02
*KCND3*	0.16 (0.06–0.41)	6.94E−06	2.89E−03	0.18 (0.08–0.43)	1.49E−06	1.30E−03
*KLK5*	4.31 (1.44–12.92)	9.99E−05	1.42E−02	7.87 (3.21–19.28)	4.58E−05	1.08E−02
*KLK6*	7.1 (2.69–18.75)	1.77E−07	3.14E−04	7.08 (2.63–19.07)	9.63E−05	1.56E−02
*KLK7*	2.02 (0.73–5.58)	3.29E−04	2.67E−02	5.09 (1.64–15.74)	7.70E−04	4.83E−02
*KRT16*	13.31 (5.01–35.38)	2.18E−04	2.17E−02	10.61 (4.52–24.91)	1.23E−05	5.05E−03
*LEMD1*	18.41 (6.93–48.86)	4.40E−07	5.85E−04	29.25 (10.64–80.37)	5.60E−06	3.39E−03
*LINC00839*	7.89 (3.39–18.4)	1.76E−06	1.44E−03	23.16 (8.68–61.83)	8.78E−05	1.50E−02
*LOXL4*	2.79 (1.01–7.71)	7.47E−04	3.96E−02	3.66 (1.46–9.18)	7.27E−05	1.38E−02
*MAP2*	4.51 (1.89–10.75)	1.09E−03	4.83E−02	4.01 (1.62–9.95)	5.57E−05	1.18E−02
*MIA*	3.96 (1.73–9.1)	5.60E−04	3.41E−02	3.57 (1.53–8.29)	8.25E−04	5.00E−02
*MSLN*	28.12 (7.23–109.38)	4.33E−04	3.00E−02	38.82 (13.62–110.7)	2.95E−06	2.19E−03
*NDRG1*	5.56 (2.28–13.56)	3.09E−04	2.64E−02	6.17 (2.74–13.92)	3.71E−04	3.30E−02
*OPRK1*	26.82 (7.14–100.76)	1.94E−04	2.08E−02	6.91 (2.28–20.91)	7.48E−06	4.13E−03
*PKP1*	13.22 (4.3–40.62)	4.46E−08	1.18E−04	4.21 (1.78–9.91)	4.22E−04	3.58E−02
*POU4F1*	11.38 (2.84–45.66)	2.17E−04	2.17E−02	16.17 (5.44–48.02)	1.06E−06	1.12E−03
*RNF150*	5.72 (2.4–13.6)	6.63E−05	1.09E−02	13.78 (5.61–33.81)	5.37E−05	1.16E−02
*SIRT5*	3.77 (1.47–9.69)	2.00E−04	2.08E−02	3.17 (1.35–7.47)	2.44E−04	2.66E−02
*SIX3*	2.32 (0.69–7.88)	1.75E−04	1.96E−02	10.31 (2.83–37.61)	4.55E−04	3.69E−02
*SOX6*	12.46 (4.51–34.46)	1.16E−04	1.58E−02	18.88 (7.65–46.58)	2.35E−08	9.23E−05
*SOX8*	5.8 (1.71–19.69)	1.07E−04	1.50E−02	15.5 (4.7–51.09)	1.71E−04	2.23E−02
*STAC*	10.58 (4.12–27.16)	3.62E−07	5.13E−04	9.14 (3.6–23.17)	4.82E−07	7.44E−04
*SYT9*	0.14 (0.04–0.44)	4.44E−04	3.02E−02	0.07 (0.02–0.21)	5.33E−04	3.92E−02
*TDRD6*	4.44 (1.86–10.62)	4.21E−04	2.93E−02	5.14 (1.99–13.29)	6.65E−05	1.28E−02
*VGLL1*	6.41 (2.77–14.87)	7.15E−05	1.14E−02	35.59 (14.02–90.34)	2.86E−04	2.89E−02

A ratio > 1 implies greater gene expression variability in *BRCA1*-associated tumours

### EN1 gene expression variability

We used the publically available METABRIC dataset [42] to further interrogate *EN1* gene expression in 1904 breast tumours (Fig. 2C, D). Consistent with analysis of the four breast cancer microarray datasets, basal-like breast tumours had significantly greater *EN1* expression variability (Fig. 2C, *p* = 3.7 × 10^−146^). Additionally, as *BRCA1*-associated breast tumours are typically oestrogen receptor (ER) negative we explored the correlation with *ESR1*. ER negative tumours had significantly greater expression variability (Fig. 2D, Brown–Forsythe test *p* = 2.19 × 10^−106^) and tumours with low *ESR1* expression had a greater range of expression. Lastly, to determine if gene dosage was the cause of variable expression we measured the correlation of copy number with *EN1* expression. There was no correlation between copy number and gene expression (*r*^2^ = 0.026) or expression variability (*r*^2^ = 0.023, Supplementary Fig. 3).

To assess intra-tumoural expression of *EN1*, breast tumour tissue microarray cores from 503 patients were assayed using RNA in situ hybridisation. These samples included tumour tissue from 151 *BRCA1*-associated and 124 *BRCA2*-associated breast cancer cases (Supplementary Tables 2, 4). Tissue microarrays were stained and scored for the abundantly expressed *PPIB* and a negative control targeting *dapB*. Tumour cores with scores > 2 for the *PPIB* and 0 for *dapB* were considered high quality and used to investigate *EN1* expression. Two-hundred-and-thirteen tumours had positive *PPIB* mRNA signals with 141/213 tumours being scored 2 or greater for *PPIB* staining. No *dapB* signal was detected in any tumours.

**Fig. 3:**
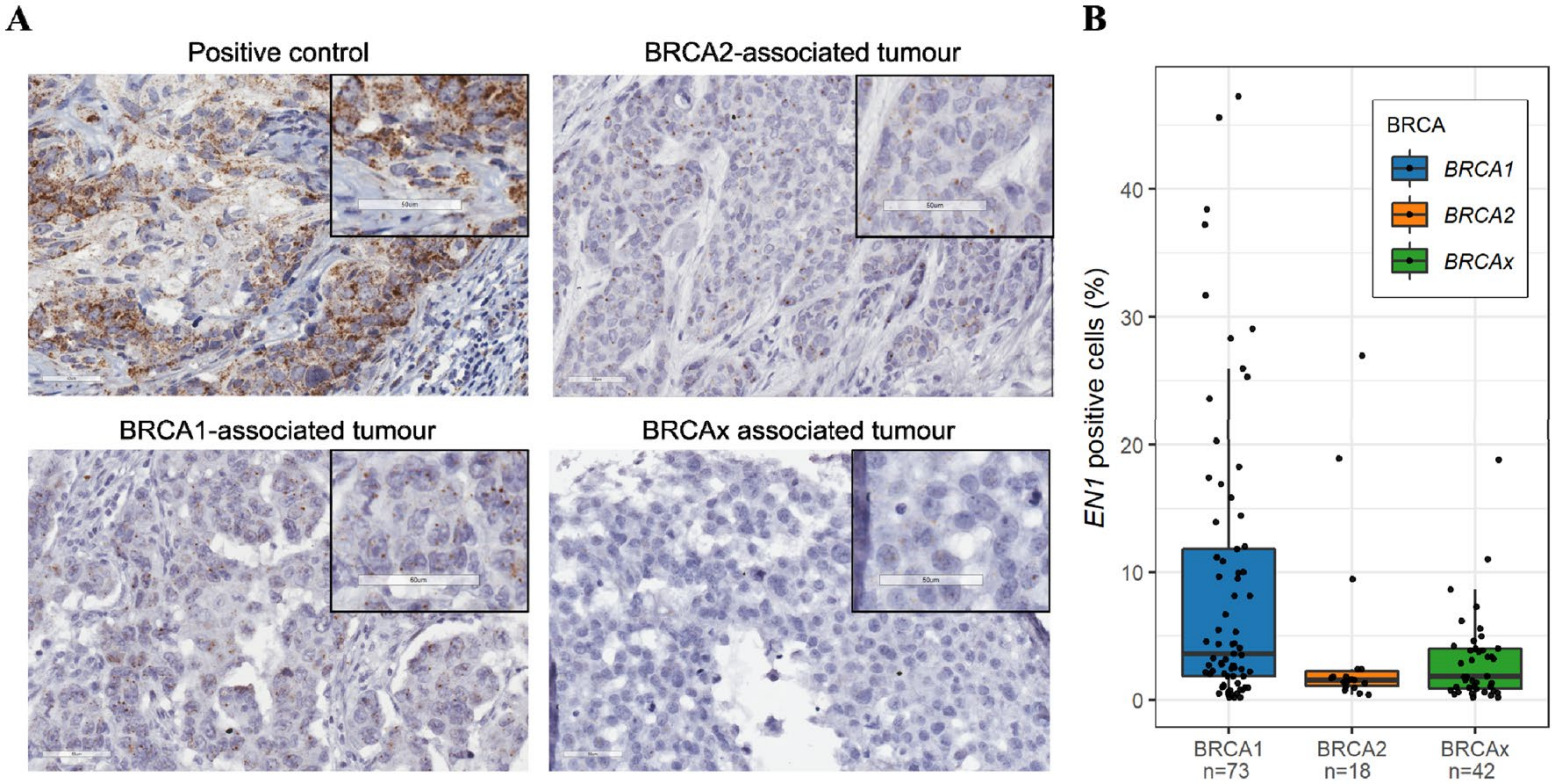
*EN1* RNAscope® of breast tumour cores. **A** Representative images of *BRCA1*-, *BRCA2*- and non-*BRCA1*/2-associated breast tumours stained for *EN1* and a section stained with the positive control probe (*PPIB*). **B** Percentage of *EN1*-positive tumour cells in each familial tumour type. A significant difference (Brown–Forsythe test) in *EN1* expression variability was observed between *BRCA1*-associated and BRCAx tumours

The LEICA RNA ISH Algorithm was used to estimate the abundance of RNA signals and the percentage of positively stained cells. Consistent with our transcriptome analysis, in situ expression analysis of *BRCA1*-associated tumours showed significantly greater *EN1* expression variability (Fig. 3, *p* = 6.7 × 10^−04^).

### Transcription regulators enrichment analysis

**Fig. 4:**
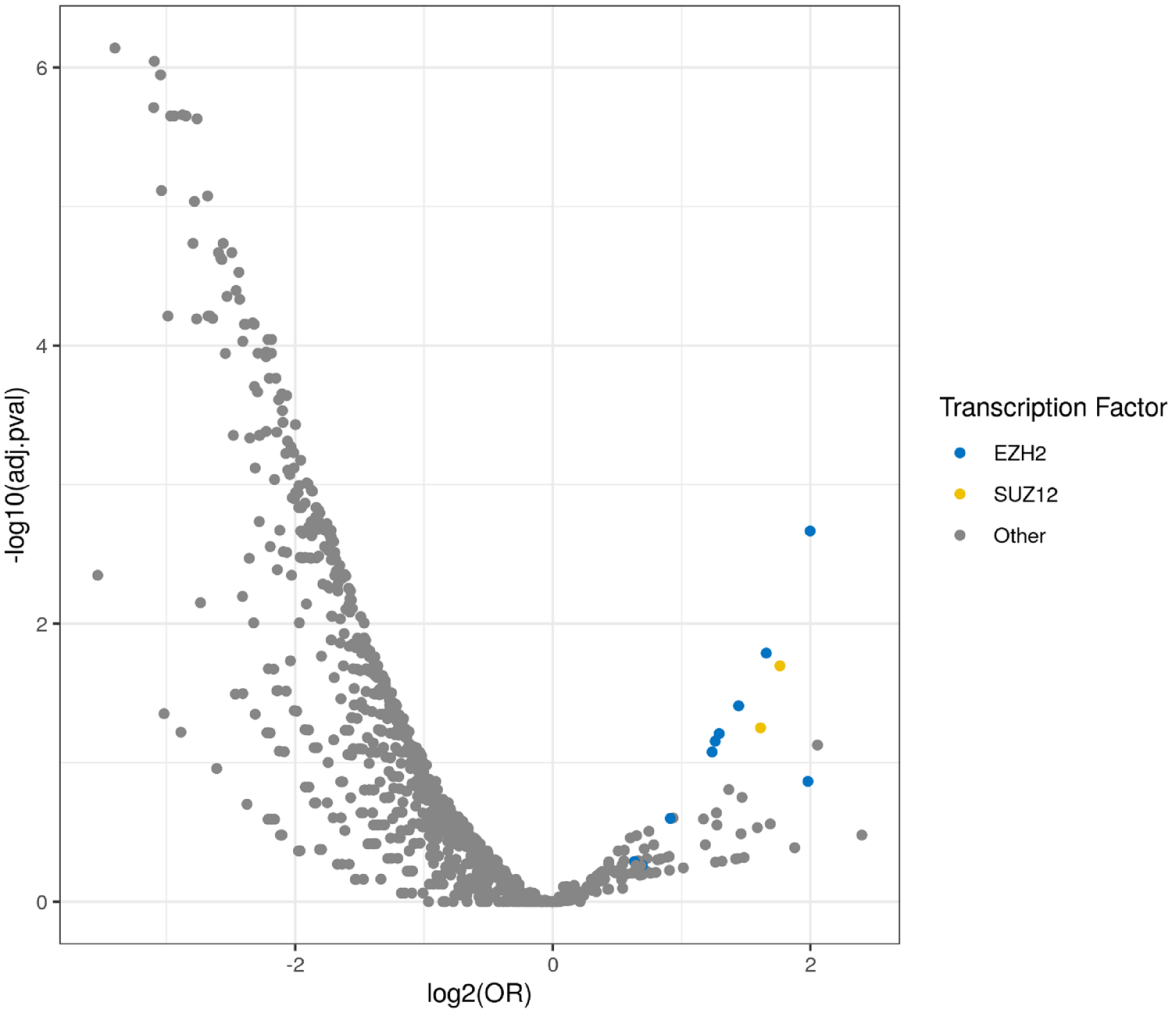
Transcription regulators enrichment analysis for *BRCA1* DV genes. Each dot represents significant over/underrepresentation of a single ChIP-Seq dataset. Log odds ratio (OR) > 0 indicates overrepresentation of *BRCA1* DV genes in a ChIP-Seq dataset. Polycomb repressive complex 2 components, EZH2 (blue) and SUZ12 (yellow) are associated with *BRCA1* DV genes

**Fig. 5:**
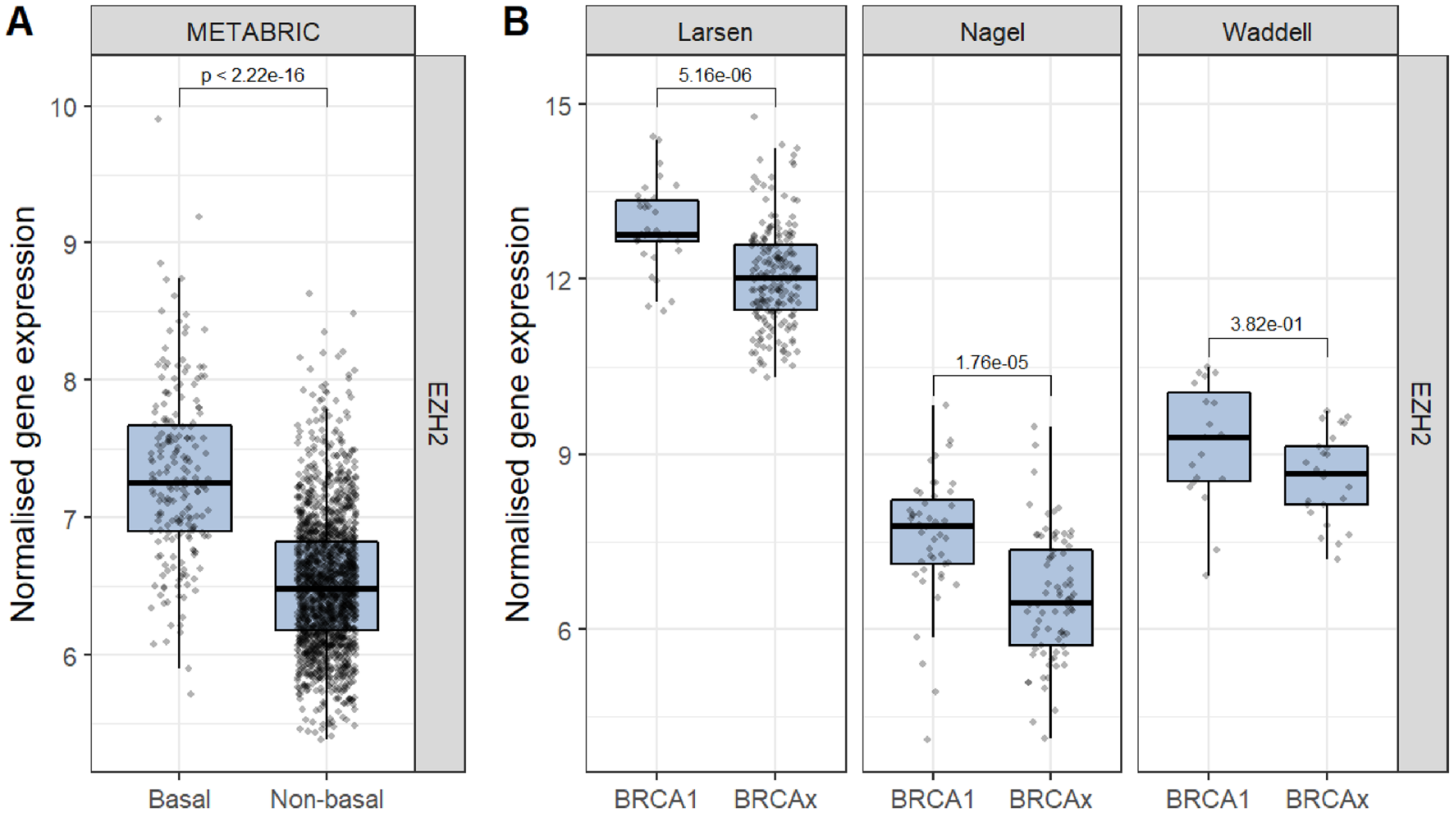
*EZH2* expression in breast tumours stratified by basal-like molecular subtype (**A**) or *BRCA1* (**B**) status. The difference of expression was calculated using the Welch *t* test

To identify transcription regulators that may be responsible for increased *BRCA1*-associated expression variability, we used the TFEA.ChIP package in R to identify transcription regulators overrepresented in the 40 *BRCA1*-associated DV genes (Table 2). Firstly, we identified individual ChIP-seq datasets associated with *BRCA1*-associated DV genes (Fig. 4) and then summarised these results at the transcription regulator level in order to identify transcription regulators that are enriched or depleted within *BRCA1*-associated DV genes (Supplementary Table 3). The top ranked ChIP-seq dataset in human mammary epithelial cells (HMEC) was associated with *EZH2* as shown in (Supplementary Table 3). Further, *EZH2* was the top ranked transcription regulator associated with *BRCA1*-related DV genes (Supplementary Table 3). Interestingly, *EZH2* was significantly overexpressed in *BRCA1*-associated and basal-like breast tumours (Fig. 5).

## Discussion

The robust and replicable nature of gene expression measurements provides an excellent opportunity to investigate biological variability [11]. We explored publicly available familial breast cancer microarray datasets for phenotypes associated with *BRCA1*- and *BRCA2*-related breast tumours. In addition, a cohort of 2116 breast tumours profiled on Affymetrix microarray platforms was included to identify gene expression variability in basal-like tumours. In this study, we identified that *BRCA1*-associated and basal-like breast tumours displayed greater gene expression variability compared to non-*BRCA1* and non-basal tumours, respectively. These observations were consistent across all microarray datasets explored and across three different measures of variability. Furthermore, no significant transcriptome-wide changes in mean expression were observed between any tumour groups. Together these results suggest that globally expression variability associates with phenotypic features of *BRCA1-*associated cancer to a stronger degree than mean expression and at an individual gene level, associations may not be statistically significant if only mean expression level is examined.

A number of studies have used transcriptome variability in humans to described differences in disease and development states [12, 14, 16–18]. One study by Bueno and Mar [18] has explored gene expression variability to identify synthetic lethal genes associated with *BRCA2* loss-of-function ovarian tumours. The authors proposed that 54 stably expressed (low variable) genes in *BRCA2*-related tumours may be essential in *BRCA2*-related tumour viability. However, there was no formal statistical test that estimated the association of gene expression variability. Our analysis in *BRCA2*-associated breast tumours suggested that no genes were differentially variable. However, it is possible that the small number of *BRCA2* samples in the microarray datasets hindered the identification of *BRCA2*-associated DV genes.

Transcriptome-wide gene expression variability may be driven by tumour heterogeneity, aggressiveness and cellular content. For example, more aggressive lymphocytic leukaemias are associated with greater gene expression variability [17], an observation that is consistent with the greater gene expression variability seen in *BRCA1*-associated and basal-like breast tumours. Furthermore, the epigenetic status of cells is a heritable trait that also has significant variability [43]. Specifically, DNA methylation can contribute to gene expression variability [44] and the methylation status of tumour cells may influence gene expression variability described in this study. Consistent with this, the top ranked transcription regulator associated with 40 *BRCA1* DV genes was EZH2, a component of the Polycomb repressive complex 2 (PRC2). PRC2 is important for H3 lysine 27 trimethylation (H3K27me3) and the stable repression of transcription [45]. *EZH2* has been shown to be overexpressed in a number of tumours including *BRCA1*-deficient breast cancers [45, 46]. In addition, loss of BRCA1 function and the decrease in *BRCA1* expression can alter the occupancy of EZH2 on DNA and increase H3K27me3 levels [47]. These *BRCA1*-related changes in epigenetic regulation are potential mechanisms that may alter transcriptional control, ultimately leading to increased cellular gene expression variability.

Technical variation is expected to contribute randomly to each sample; however, as there was no standardised protocol between and within the datasets used, it is plausible that processes such as sample collection and RNA extraction may influence variability. By considering only genes that were variably expressed across all microarray datasets we are able to reduce false-positive associations induced by these types of experimental artefacts. Our approach identified two genes (*EN1* and *IGF2BP3*) that had increased variability in *BRCA1*-associated breast tumours. *EN1* encodes the transcription factor Engrailed Homoeobox 1 and has been extensively investigated in neuronal development. Ectopic *EN1* expression improves neurons’ survival and protects against apoptosis [48]. Conversely, knockdown of expression in dopaminergic neurons has been shown to induce apoptosis [49]. *EN1* has been observed to be overexpressed in triple-negative breast cancers and basal-like breast cancers [50–52]. SDs and inter-quartile ranges reported by these studies were suggestive of increased gene expression variability similar to that seen in this study; however, these were not formally tested. Additionally, *EN1* expression has been associated with poorer survival and a greater probability of brain metastases. Interestingly, reduced expression of *EN1* in basal, but not luminal, breast cancer cell lines decreases viability, which can be partially rescued by overexpression of *EN1* [52]. The evaluation of EN1 protein by immunohistochemistry has been perplexing, with Kim and colleagues previously describing EN1 protein expression as being associated with improved survival in triple-negative breast cancer, opposite to that of *EN1* RNA [51]. The discrepancy may in part be due to poor antibodies targeting EN1 [52] or post-transcriptional processes. In this study, we have investigated the utility of RNAscope to measure gene expression in situ and we were able to recapture the inter-tumour variability observed in the microarray analysis. The implementation of RNA in situ hybridisation can overcome the issue of antibody specificity and may help facilitate replication of survival trends. However, the lack of survival data limited our ability to formally test for associations. Currently, measurement of intra-tumoural variability remains challenging, particularly due to the discrimination of signal in dark-stained chromatin and granulated nuclei. The development of more sophisticated algorithms in the future may provide greater power to assess variability within individual sections and allow the ability to test for associations with clinicopathological data. Furthermore, to fully appreciate tumour variability, complete tumour sections may be required for future studies.

## Conclusion

*BRCA1*-associated and basal-like breast tumours displayed a phenotype of greater gene expression variability, with no change in global RNA abundance. *EN1* had greater expression variability in *BRCA1*-associated breast tumours, and this was captured in transcriptomic and RNA ISH analyses. We conclude that the expression variability of a gene is replicable, thereby laying a foundation for future studies aiming to better understand the molecular mechanisms underlying the development of basal-like breast tumours.

## Supplementary Information

Below is the link to the electronic supplementary material.

Supplementary file1 (PDF 893 kb)

Supplementary file2 (XLSX 154 kb)

Supplementary file3 (XLSX 23 kb)

## Data Availability

Original mircoarray datasets (GSE19177, GSE27830 and GSE49481) can be found in https://www.ncbi.nlm.nih.gov/geo/. The METABRIC dataset [42] was accessed through cbioportal (https://www.cbioportal.org/). The meta-cohort has been previously described [33, 34].

## Abbreviations

SD: Standard deviations
CV: Coefficient of variance
MAD: Median absolute deviation
RNA ISH: RNA in situ hybridisation
ER: Oestrogen receptor
DV: Differentially variable
