# Supplementary material for: Increased gene expression variability in BRCA1-associated and basal-like breast tumours
Source: Breast Cancer Res Treat. 2021 Jul 21;189(2):363–75. doi: 10.1007/s10549-021-06328-y (PMC8357684; doi:10.1007/s10549-021-06328-y)
Supplement: Supplementary file 1 — Supplementary file1 (PDF 893 kb) [file 10549_2021_6328_MOESM1_ESM.pdf]

Increased gene expression variability in *BRCA1*-associated and basal-like breast tumours

Wiggins, George A R[1], Black, Michael A[2], Dunbier, Anita[2], Morley-Bunker, Arthur E[1], kConFab Investigators[3,4], Pearson, John F[1,5#], Walker, Logan C[1#*]

[1]*Department of Pathology and Biomedical Science, University of Otago Christchurch, NZ.*

[2]*Department of Biochemistry, University of Otago Dunedin, NZ.*

[3]*Sir Peter MacCallum Department of Oncology, University of Melbourne, Melbourne, VIC, Australia*

[4]*Research Department, Peter MacCallum Cancer Center, Melbourne, VIC, Australia*

[5]*Biostatistics and Computational Biology Unit, University of Otago Christchurch, NZ.*

[#]*Contributed equally*

[*]*Corresponding author:* Logan C. Walker

Tel: +64 3 364 0544

Email: logan.walker@otago.ac.nz

Supplementary Table 1. RNAscope scoring criteria

Score	Criteria
0	No punctuated signal
0.5	0-30% of cells with 1-3 punctuate signals/cell
1	>30% of cells with 1-3 punctuate signals/cell
2	4-9 punctuate signals/cell with no clustering
3	10^+ punctuate signals/cell with <10% of signals clustering
4	10^+ punctuate signals/cell with >10% of signals clustering

Supplementary Table 2. Clinicopathological data for breast tumours used for RNAscope analysis

	TMA8	TMA9	TMA10	TMA11	TMA12	TMA16	TMA17
N	60	60	60	57	24	121	121
BRCA status							
BRCA1	60	-	60	-	24	4	3
BRCA2	-	60	-	57	-	3	4
ER status							
Positive	12	30	7	25	1	60	51
Negative	34	7	34	11	10	19	23
Unknown	14	23	19	21	13	42	47
PR Status							
Positive	11	25	8	23	2	56	43
Negative	34	23	30	10	4	18	20
Unknown	15	12	22	24	18	47	58
HER2							
Positive	4	5	3	4	1	7	13
Negative	19	12	14	3	1	14	20
Unknown	37	43	43	50	22	100	88
CK5							
Positive	31	8	27	5	13	10	8
Negative	25	48	26	41	9	94	88
Unknown	4	4	7	11	2	17	25

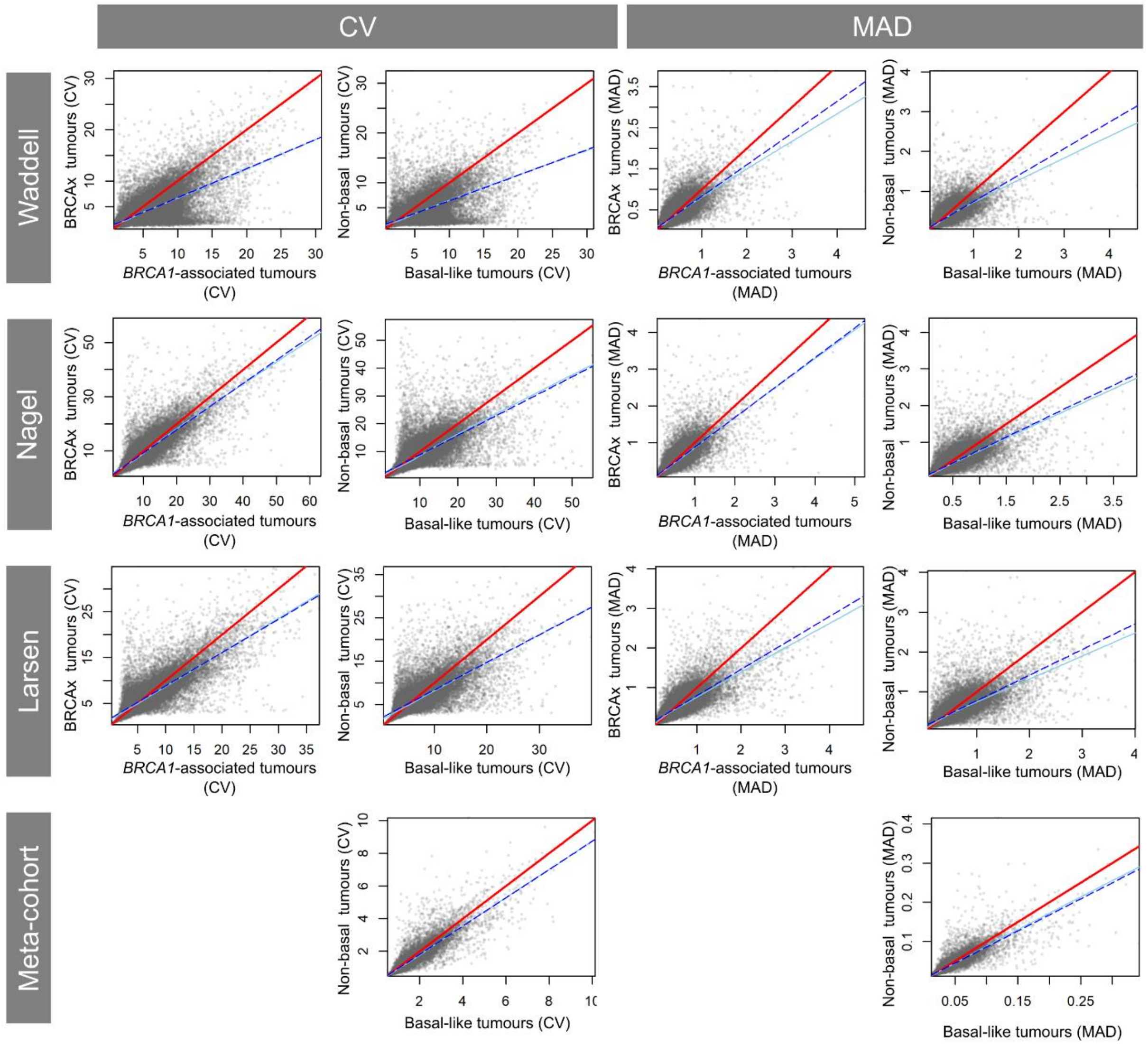

Supplementary Fig 1. Transcriptome-wide gene expression variability in breast tumours as measured by gene-specific CV and MAD. *BRCA1*-associated and basal-like breast tumours each show greater gene-specific CV and MAD values compared to BRCAx and non-basal tumour, respectively. A model of equity (red line) was compared to the linear model (blue dashed line) and polynomial regression (sky blue line).

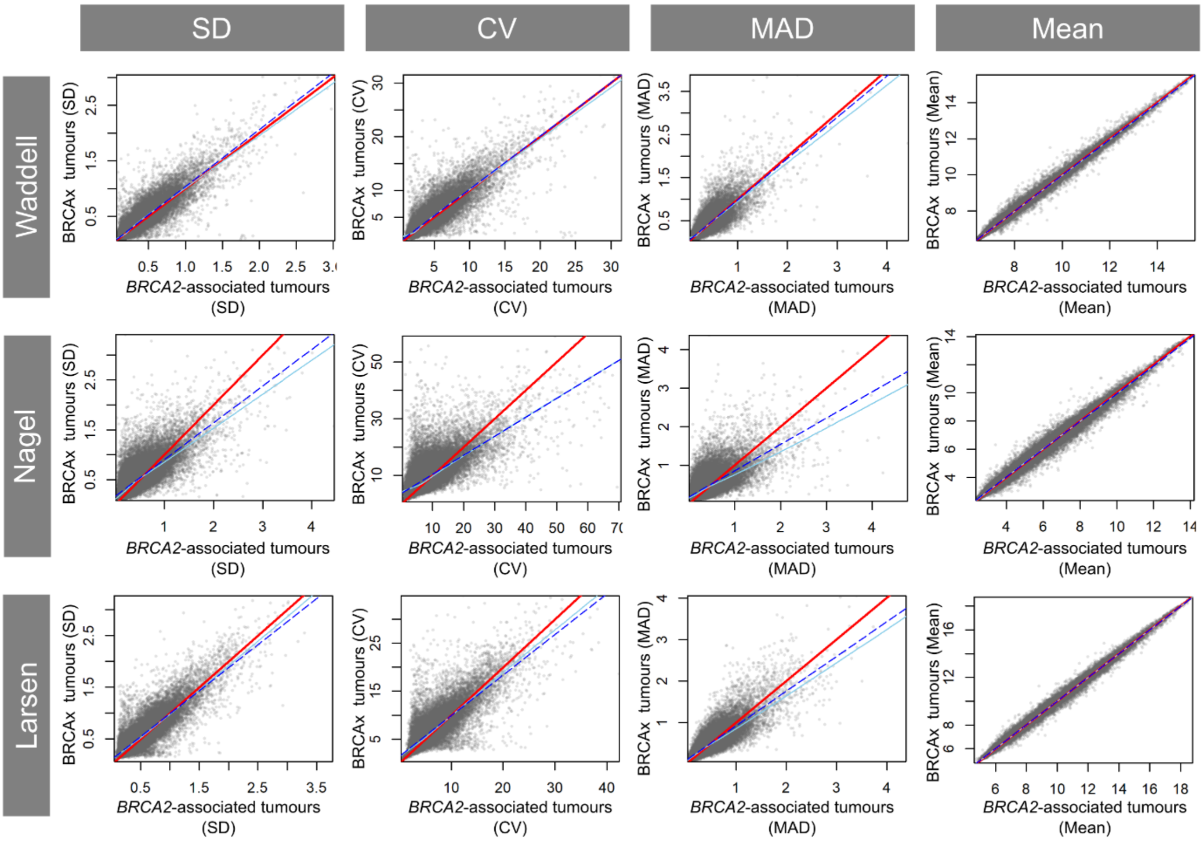

Supplementary Fig 2. Transcriptome-wide gene expression variability in breast tumours as measured by gene-specific SD, CV and MAD. *BRCA2*-associated gene expression variability is inconsistent compared to BRCAx across the three microarray datasets. In contrast, global gene-specific means between tumour groups are comparable. A model of equity (red line) was compared to the linear model (blue dashed line) and polynomial regression (sky blue line).

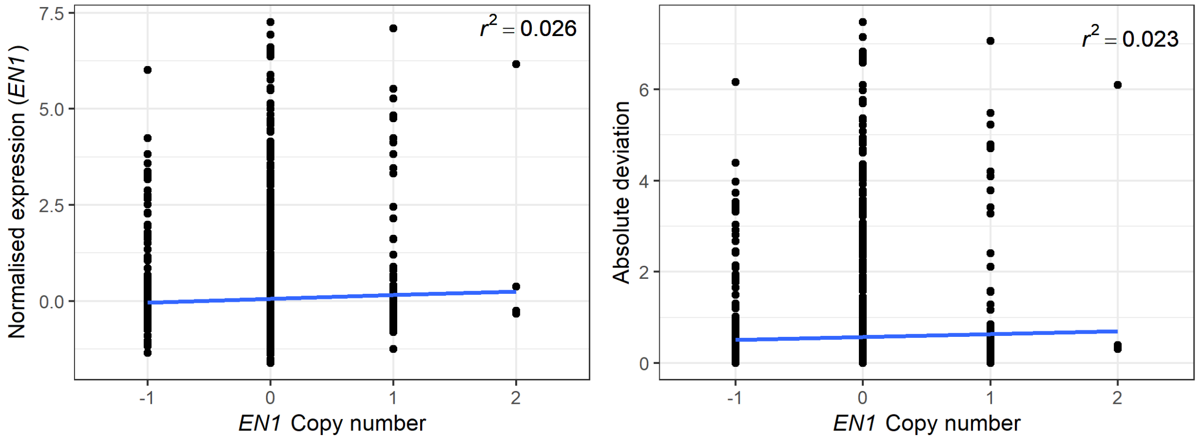

Supplementary Fig 3. Correlation of *EN1* copy number with *EN1* gene expression (left) and *EN1* variability (right). Variability is described as the absolute deviation from the median within each copy number status. The linear model (blue) describes the strength of correlation
